# Synthesis and applications of bimetallic metal–organic frameworks catalyst for activating peroxymonosulfate for carbamazepine degradation

**DOI:** 10.3389/fchem.2026.1891127

**Published:** 2026-08-28

**Authors:** Xin Zhong, Qi Huang, Meihuan Ji, Zhao Liao, Chaosheng Chen

**Affiliations:** 1 Experimental Teaching Platform, Beijing Normal University at Zhuhai, Zhuhai, China; 2 Faculty of Arts and Sciences, Beijing Normal University, Zhuhai, China

**Keywords:** antibiotics, bimetallic MOFs, carbamazepine, pathways, PMS activation

## Abstract

In this research, a series of bimetallic Cu_x_Zn_y_-MOFs catalysts was constructed via a convenient solvothermal synthesis procedure by using the 2,3,6,7,10,11-hexaiminotriphenylene (HATP) ligand with copper and zinc metal ions. Among the series catalysts, Cu_2_Zn_1_-MOFs exhibited outstanding adsorption efficiency and degradation performance, and achieved 97.83% carbamazepine (CBZ) removal within 15 min using a catalyst dosage of 0.05 g L^-1^ and 0.5 mM peroxymonosulfate (PMS) concentration. The catalytic degradation rate constant of CBZ by Cu_2_Zn_1_-MOFs catalyst reached to 0.1833 min^-1^, which is 3.1 times higher than Cu-MOFs. In order to investigate the catalytic mechanism, density functional theory (DFT) calculations and experimental results were conducted which revealed the enhanced adsorption efficiency due to the incorporation of zinc into Cu-MOFs, thereby promoting the generation of reactive oxygen species in PMS activation. Furthermore, the CBZ degradation pathway was elucidated by detected the intermediates using LC/MS during the oxidation process. These findings highlighted the synergistic effects of bimetallic MOFs as highly efficient heterogeneous Fenton–like catalyst, which could expand the potential actual application.

## 1 Introduction

Pharmaceuticals and personal care products (PPCPs) have been paid great attention and raised lots of concerns due to their increasing consumption, growing continuous release and potential ecological risks to aquatic ecosystems (Chen et al., 2023; Kumar et al., 2020; Zhou et al., 2020). These pollutants are characterized by high persistence, strong bio–accumulation potential, and inherent ecotoxicity, which posed a significant challenge to conventional water treatment technologies (Sun B. et al., 2026; Jia et al., 2024; Liu B. et al., 2026). Carbamazepine is a widely used pharmaceutical chemical for the treatment of epilepsy, which has been classified as a priority pollutant by the European Union (EU) and the U.S. Environmental Protection Agency, attributed to its notable environmental risk and potential effect to the ecosystems (Zheng et al., 2024). Therefore, extensive efforts have been made to addressing the challenge and removing similar kinds of organic contaminants from aquatic environment. Among various water treatment technologies, sulfate radical–based advanced oxidation processes (SR–AOPs) have been demonstrated to exhibit distinct superiority in the elimination of refractory organic pollutants in aqueous solution (He et al., 2014; Jackulin et al., 2024; Yang et al., 2025). Through activating peroxymonosulfate (PMS) or peroxydisulfate (PDS) to generate reactive oxygen species, SR–AOPs achieve high catalytic activities, benefiting from high redox potential, extended radical half–life and stable catalytic activity over a wide pH range (Chen et al., 2026). Therefore, SR–AOPs exhibit favorable applicability and degradation performance in actual water treatment, offering a promising technology for remediation of emerging aquatic contaminants.

The degradation performance of SR–AOPs depends on the development of efficient peroxymonosulfate (PMS) activation process. Compared with PDS (S_2_O_8_^2-^), PMS (mainly HSO_5_^−^) is considered to be more active due to its asymmetric structure with long superoxide bond (I_o_–_o_ = 1.326 Å) (Kohantorabi et al., 2021; Li et al., 2023; Zhang M. et al., 2026). Many efforts were made to employ different transition metal oxides for PMS activation, which exhibited enlarged specific surface areas and active sites and improved catalytic performance (Allaoui et al., 2026; Ma et al., 2026; Wu W. et al., 2025). However, metal–organic frameworks (MOFs) have been considered as a highly promising research hotspot, driven by the high specific surface area and facile synthesis accessibility (Liu F. et al., 2026). In general, extensive efforts have been devoted to addressing this challenge by constructing polynuclear active sites, which have been proven to exhibit efficient catalytic performance (Park et al., 2025). Benefiting from synergistic interaction between metal active sites, bimetallic MOFs have emerged as highly promising candidates, offered superior catalytic activity and enlarged specific surface area compared to monometallic MOFs–based catalysts, thus accelerating the degradation kinetics of target pollutants in the solution (Li et al., 2022; Zareen et al., 2025). Transition metals, including Co, Fe, Mn, Cu, Zn as well as their corresponding oxides, are widely recognized as efficient catalysts for PMS activation. Owing to their strong redox capability, efficient electron transfer was promoted and underwent mild operation conditions during the oxidation process (Bian et al., 2025; Huang et al., 2026; Pang et al., 2025; Tan et al., 2022; Xie et al., 2023).

Monometallic MOF catalysts derived with one type of metal elements have been regarded as insufficient to meet the demands for practical applications, owing to the intrinsic limitations in structural stability and catalytic activity (Guo et al., 2025; Liu et al., 2025; Liu Y. et al., 2026). Recent research has demonstrated that mixed metal oxides and bimetallic MOF catalysts can effectively enhance the catalytic performance (Liu F. et al., 2026; Lu J. et al., 2025; Zhang L. et al., 2025; Zhao et al., 2025). Benefiting from the synergistic interaction between metal ions, enhanced catalytic activity and optimized structural properties can be achieved, revealing great potential for efficient pollutant elimination (Wang et al., 2025). Li et al. reported that Co–Ni bimetallic MOFs can achieve complete removal of SMX within 7 min, which was better than in monometallic MOFs (Li M. et al., 2025). Moreover, Shi et al. synthesized a series of Cu–Co bimetallic MOFs for PMS activation, with the addition of Co doping, the catalytic performance was improved and achieved completely PNP removal in 30 min (Shi et al., 2025). Zhang et al. synthesized zinc–cobalt bimetallic metal organic framework used to activate peroxymonosulfate (PMS) for the removal of ACE, which achieved 96.93% in 90 min (Zhang X. et al., 2026). The superior catalytic performance of bimetallic MOFs can be ascribed to the synergistic effect that provides stronger adsorption capacity towards PMS molecules (Li et al., 2025a). Accordingly, incorporating different transition metals into MOFs catalyst to construct bimetallic MOFs structures represents a feasible strategy to achieve high catalytic activities.

In this work, a series of Cu_x_Zn_y_-MOFs was synthesized via a solvothermal method and employed efficient heterogeneous catalyst for PMS activation. As a bimetallic ion doped MOFs catalyst, Cu_x_Zn_y_-MOFs exhibits an improvement of exposed active sites. In the meanwhile, the synergistic interaction between copper and zinc was found to remarkably promote the catalytic activity. The catalysts were characterized before and after the reaction through different characterization technology, including XRD, SEM, TEM/EDS, XPS and FTIR, to reveal the possible degradation mechanism in the Cu_x_Zn_y_-MOFs/PMS system. The effect of key parameters on the degradation efficiency of CBZ was systematically investigated, including catalyst dosage, PMS concentration, initial pH and inorganic ions. The stability and reusability of the catalysts was also evaluated. Furthermore, a predicable degradation pathway of CBZ was proposed according to the intermediates identified during the degradation process. The catalytic behavior toward CBZ molecules was systematically investigated by density functional theory (DFT) calculations. This work offers a different insight into the structural regulation of bimetallic MOFs catalysts, which could be served as a new strategy for the precise identification of active sites in Fenton–like process. The highlights of this study are that bimetallic Cu_x_Zn_y_-MOFs exhibit outstanding carbamazepine (CBZ) removal efficiency and rapid degradation kinetics, the dominant reactive oxidative species (ROS) driving pollutant degradation via accelerated ROS production owing to bimetallic doping. This study provided meaningful guidance for exploring reactive oxidation process degradation pathway and possible catalytic mechanism in the SR–AOPs, promoting the catalyst design and practical application for environmental remediation, which show limited metal leaching.

## 2 Experimental conditions

### 2.1 Reagents and chemicals

The reagents and chemicals required for the experiment are listed in the Supporting Information (Text S1, Supporting Information).

### 2.2 Catalyst synthesis procedure

The catalyst synthesis procedure required for the degradation experiment are listed in the Supporting Information (Text S2, Supporting Information).

### 2.3 Catalyst characterization

The characterization instruments required for the degradation experiment are shown in the Supporting Information (Text S3, Supporting Information).

### 2.4 Experimental procedure

The experimental procedure required for the degradation experiment are shown in the Supporting Information (Text S4, Supporting Information).

## 3 Results and discussion

### 3.1 Characterization of Cu_x_Zn_y_-MOFs catalysts

**FIGURE 1:**
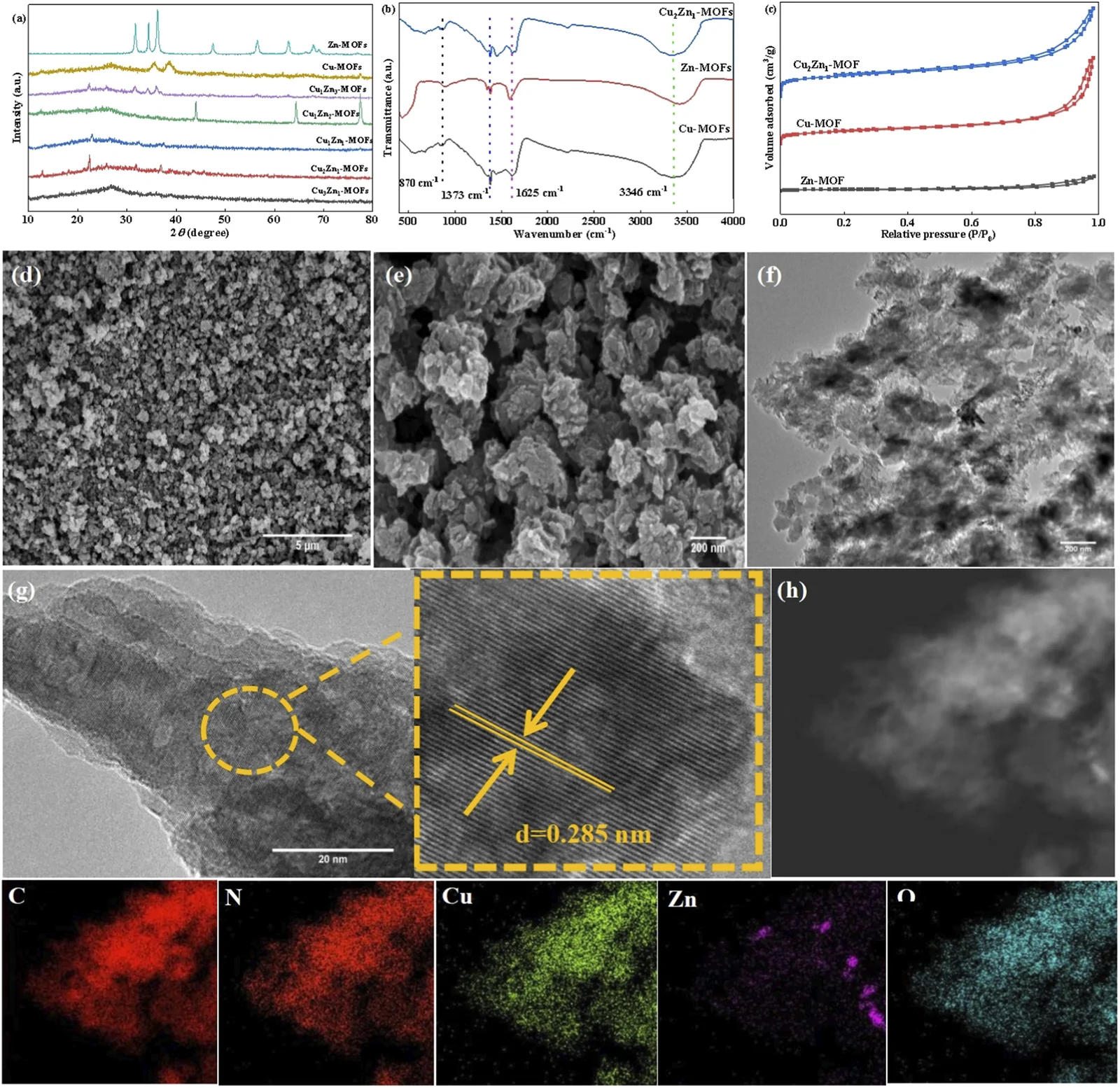
**(a)** XRD patterns of synthesized Cu_x_Zn_y_-MOFs catalyst; **(b)** FTIR spectra and adsorption-desorption isotherms **(c)** of Cu-MOFs,Zn-MOFs and Cu_2_Zn_1_-MOF scatalysts; **(d,e)** SEM images of Cu_2_Zn_1_-MOFs; HRTEM images graphs and EDS mapping **(f–h)** of Cu_2_Zn_1_-MOF scatalysts.

The crystalline phase composition of the series Cu_x_Zn_y_-MOFs catalysts was characterized by powder X–ray diffraction (XRD) characterization to identify the characteristic diffraction peaks. Figure 1a demonstrates the XRD patterns of the prepared Cu_x_Zn_y_-MOFs catalysts. It can be seen that the main diffraction peaks of Cu_x_Zn_y_-MOFs catalysts are similar to monometallic MOFs, implying the successful synthesis of bimetallic MOFs catalyst. A broad diffraction peak centered at around 27° was found in Cu-MOFs and Zn-MOFs catalyst, assignable to the (001) plane and suggesting the stacking of the two layered structure (Chen et al., 2020; Peng X. et al., 2025). With the incorporated Cu and Zn ions, the diffraction peak intensity and crystalline was reduced, which demonstrates that the introduction of copper and zinc ions compromises the structural order of MOFs. The results demonstrated that Cu_x_Zn_y_-MOFs possessed similar two–dimensional porous structure with monometallic MOFs, whereas the lattice disturbance originating from the ionic radii and coordination behaviors of Cu^2+^, where the incorporation of Zn^2+^ ions may also affect the crystalline of Cu-MOFs.

The FTIR spectrum displayed similar absorption bands at 3,346 cm^-1^, corresponding to the stretching vibration of primary amine doublets–NH_2_ and the vibration peak of the–NH group in the benzene ring (Nazira et al., 2021), as shown in Figure 1b. The sharp stretching peak at 1,373 cm^-1^ could be assigned to C–N groups. The results showed that the catalyst was successful coordinated of metal ions with HATP ligand. The absorption bands at 1,625 cm^-1^ and 870 cm^-1^ correspond to aromatic C=C stretching and C–H bending vibrations, respectively. Moreover, the textural properties of Cu-MOFs, Zn-MOFs, and Cu_2_Zn_1_-MOFs were characterized including specific surface area (BET) and pore size distribution, as shown in Figure 1c. The BET surface areas were determined to be 92.2, 8.6, and 157.2 m^2^/g, with corresponding pore volumes of 0.28, 0.05, and 0.31 cm^3^/g of Cu-MOFs, Zn-MOFs, and Cu_2_Zn_1_-MOFs, respectively. The significantly enhanced surface area of the bimetallic MOFs facilitates greater exposure of active sites (Li et al., 2025b). Figures 1d,e illustrates the morphological characteristics of Cu_2_Zn_1_-MOFs catalyst. For bimetallic Cu_2_Zn_1_-MOFs, flower–like morphology was observed transitioned between two distinct forms. The TEM images and EDS mapping showed the structural and compositional for the Cu_2_Zn_1_-MOFs. The clear lattice fringes were observed in Cu_2_Zn_1_-MOFs, and the d–spacing was 0.285 nm matched the (200) lattice plane (Xing et al., 2021). Moreover, EDS mapping images demonstrated the uniform co–distribution of Cu and Zn on the catalyst surface, providing for the homogeneous bimetallic incorporation within the MOFs framework structure.

### 3.2 Catalytic properties of Cu_x_Zn_y_-MOFs catalysts in different systems

The specific surface area and pore size distributions of Cu-MOFs, Zn-MOFs, and Cu_2_Zn_1_-MOFs were investigated by BET measurements, which was in good agreement with corresponding surface area, as shown in Supplementary Table S1 and Supplementary Figure S1. The Fourier transform infrared spectral analysis (FTIR) before and after adsorption was also conducted in Supplementary Figure S2. The peak located at 621 cm^-1^ after the adsorption was corresponded to the stretching peak of metal–O, revealing the reduction of metal valence states and the in–situ formation of metal hydroxides during the reaction (Yao et al., 2025). Moreover, the peak around 1,241 cm^-1^ was extremely highlighted after the adsorption, due to the degradation intermediates adsorbed on the catalyst surface (Zhang Q. et al., 2025). After the adsorption, the peak intensity was undermined and the peak location change was observed after the adsorption due to the adsorption of N site and–NH_2_ group of CBZ in the catalyst. The catalyst structure was considered that it can facilitate the CBZ adsorption process. In summary, the adsorption between CBZ and Cu_2_Zn_1_-MOFs catalyst mainly relies on the N and O sites derived from the structure within Cu_2_Zn_1_-MOFs during the reaction (Peng Y. et al., 2025). Moreover, the nitrogen sites could conduce the adsorption process through the interaction between hydrogen bonds and CBZ. In addition, the structure framework of Cu_2_Zn_1_-MOFs catalyst could react and absorb with CBZ, thereby boosting the adsorption capacity. The adsorption kinetics of CBZ was studied by using the pseudo–first–order kinetic model and pseudo–second–order kinetic model. As presented in Supplementary Figure S3, the pseudo–second–order model yielded a higher coefficient of determination (*R* ^2^ = 0.71) compared to the pseudo–first–order model (*R* ^2^ = 0.6), indicating it more suited for fitting pseudo–second–order model. It is considered that the pseudo–second–order kinetics is indicative of chemisorption as the rate–limiting step, the adsorption of CBZ onto Cu_2_Zn_1_-MOFs is predominantly governed by chemisorption rather than physisorption.

It is known that the adsorption process can remove pollutants but cannot achieve completely mineralization. In order to obtain further degradation and mineralization of carbamazepine (CBZ), PMS activation process (i.e., Fenton–like process) was conducted (Anh et al., 2026). The degradation efficiency of CBZ was evaluated across the other catalytic oxidation process, such as Cu_2_Zn_1_-MOFs/PDS and Cu_2_Zn_1_-MOFs/H_2_O_2_. As illustrated in Supplementary Figure S4, all the Cu_2_Zn_1_-MOFs/oxidant systems showed excellent CBZ removal efficiency, with the degradation efficiencies over 95% in 15 min. Among these systems, the Cu_2_Zn_1_-MOFs/PMS system exhibited better catalytic activity, attaining a remarkable CBZ degradation efficiency of 97.83% within a 15 min time–period. The results certified that the Cu_2_Zn_1_-MOFs catalysts can be widely applied in multiple oxidant activation systems for CBZ removal. The asymmetric structure in PMS took advantages for efficient catalyst activation compared to PDS and H_2_O_2_ (Lu T. et al., 2025). Consequently, these results clearly identify that the Cu_2_Zn_1_-MOFs catalyst can be acted as an effective catalyst for CBZ degradation in Fenton–like process.

**FIGURE 2:**
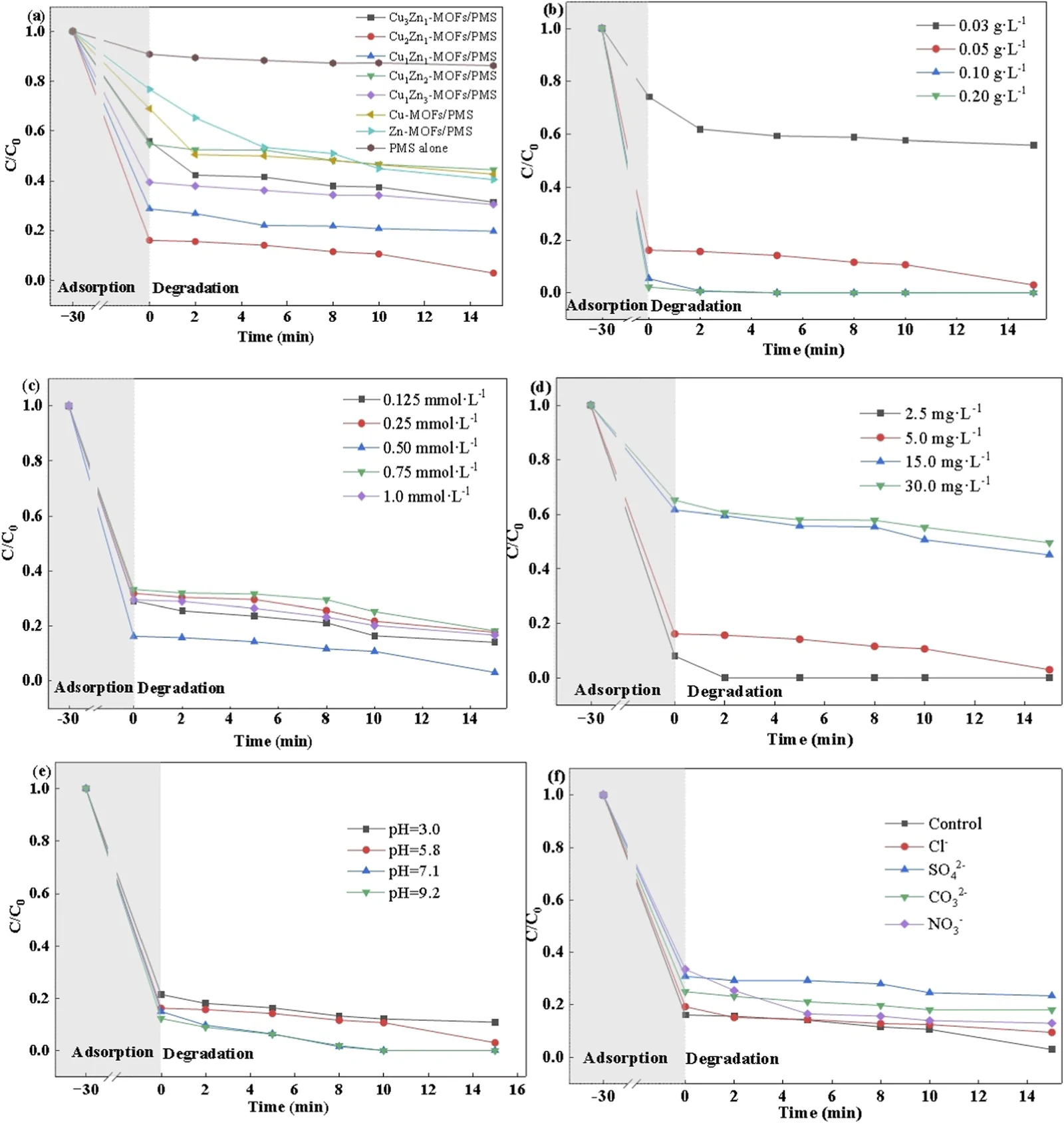
**(a)** Degradation effect of different Cu/Zn ratio in CuZny-MOFs/PMS process; **(b)** effect of Cu2Zni-MOFS catalyst dosage; **(c)** effect of PMS concentration; **(d)** effect of initial CBZ concentration; **(e)** effect of initial pH; **(f)** effect of co-existingions on CBZ removal. Conditions: CBZ 5.0 mg L^-1^; catalysts = 0.05 g L^-^¹; PMS = 0.5 mM; pH = 5.8 (unadjusted). [CI], [SO4²¯], [CO2], [NO] each at 10 mM.

As shown in Figure 2, the CBZ degradation efficiency was investigated under different reaction conditions. The process exhibited minimal oxidative capability by using peroxymonosulfate (PMS) alone, where negligible CBZ removal was achieved. The low efficiency highlighted the negligible catalytic activity of non–activated PMS, due to the limited ability to generate reactive oxygen species alone, specifically sulfate radicals (SO_4_^•-^) and hydroxyl radicals (^•^OH) (Dai et al., 2025). With the addition of peroxymonosulfate (PMS), the Cu_x_Zn_y_-MOFs/PMS systems showed much improvement in the degradation of carbamazepine (CBZ), showing their superior catalytic performance in the PMS activation process. Furthermore, the catalytic performance of Cu_x_Zn_y_-MOFs exhibited good catalytic performance which was compared with the previous studies for PMS activation, as shown in Supplementary Table S2. Owing to its exceptional activity and superior degradation efficiency, Cu_x_Zn_y_-MOFs was expected to be chosen as the representative catalyst for application–oriented investigations.

Among the synthesized Cu_x_Zn_y_-MOFs catalysts, Cu_2_Zn_1_-MOFs exhibited the highest catalytic performance (Figure 2a), and the corresponding first–order reaction rate constant is 0.1833 min^-1^ when the Cu/Zn addition ratio was 2:1, the other rate constant was shown in Supplementary Figure S5. It can be seen that the catalytic performance of Cu-MOFs was better than Zn-MOFs, which can be assigned to the excessive adsorption of CBZ leading to reduced exposed active sites. Notably, CBZ degradation efficiency demonstrated a strong positive correlation with the increase of Cu/Zn ratio, as well as the oxygen vacancy concentration within the Cu_x_Zn_y_-MOFs catalysts. This correlation strongly suggests that two metal ions composite acted as core active sites, enhancing the PMS activation. Figures 2b–f presents the catalytic performance of CBZ removal for the Cu_x_Zn_y_-MOFs/PMS process by varying experimental parameters. As shown in Figure 2b, increasing the catalyst dosage from 0.03 g L^-1^ to 0.05 g L^-1^ led to a significant enhancement in CBZ degradation efficiency. While the catalyst dosage was over 0.05 g·L^-1^, CBZ removal was completely 100%, representing an approximately 30% improvement over the performance at 0.03 g·L^-1^ catalyst dosage. However, a further increase in catalyst dosage from 0.10 g·L^-1^ to 0.20 g·L^-1^, while raising the reaction rate constant from 0.955 min^-1^ to 0.998 min^-1^, resulted in no further increase in overall degradation efficiency. The results suggested that the reaction became limited in the PMS activation process.

Accordingly, increasing the PMS concentration from 0.125 mM to 0.5 mM significantly enhanced CBZ removal efficiency, which was attributed to the higher activation availability of reactive oxidants to the target contaminant (Figure 2c). Meanwhile, a further increase to 1.0 mM resulted in an unexpected decrease in the reaction efficiency and rate. The decline is attributed to the self–quenching of excess sulfate radicals (SO_4_^•-^), as described by Equations 1–3, which reduces the number of radicals available for CBZ oxidation (Pei et al., 2026). The reaction rate showed a decrease when PMS was increased from 0.75 mM to 1.0 mM, which did not regain the level observed at the optimal 0.5 mM PMS concentration. These results can be explained by the complex radical quenching reaction. When the PMS concentrations was high, the anion (HSO_5_^−^) can react with the abundant sulfate radicals (SO_4_^•-^), yielding the radical reaction, which possesses considerably weaker oxidative capability (Sun Y. et al., 2026). This side reaction effectively affects the reactive radicals, thereby diminishing the overall oxidation efficiency.

HSO5​−→O•H+SO4​•− (1)

$$SO_{4}{\text{​}}^{•-}+SO_{4}{\text{​}}^{•-}\to S_{2}O_{8}{\text{​}}^{2-}$$ (2)

$$S_{2}O_{8}{\text{​}}^{2-}+SO_{4}{\text{​}}^{•-}\to S_{2}O_{8}{\text{​}}^{•-}+SO_{4}{\text{​}}^{2-}$$ (3)

Furthermore, the degradation efficiency was distinctly dependent on the initial CBZ concentration. With the initial CBZ concentration increased from 2.5 mg·L^-1^ to 30 mg·L^-1^, a positive correlation was observed in the CBZ removal, which indicated a proportionally high catalytic capacity (Figure 2d). The observed degradation behavior which relied on the initial CBZ concentration can be attributed to the reaction stoichiometry in the Cu_x_Zn_y_-MOFs/PMS catalytic process. In addition, with catalyst dosage fixed at 0.05 g·L^-1^ and PMS concentration fixed at 0.5 mM, the total amount of degraded CBZ is inherently limited by the PMS concentration and the in–situ generated active sites. At certain catalyst dosage and PMS concentration, the degradation oxidative capacity was also determined as the reactive oxidants and catalyst dosage were fixed. Although the initial CBZ concentration was low, the degradation efficiency does not show proportional increase. As the CBZ concentration increased to 30 mg L^-1^, the degradation efficiency declined significantly, which is likely to ascribe to the inadequate generation of active radicals.

The influence of initial pH on CBZ removal is presented in Figure 2e. In the CBZ solution with unadjusted pH (initial pH = 5.8), the degradation efficiency reached to 97.83%. Notably, CBZ (pKa = 13.9) remains predominantly in its neutral molecular form over the tested pH range (3.0–9.0), thus minimizing the influence of chemical format for the degradation process (Su et al., 2026). The CBZ removal efficiency was remained high over a wide pH range. Under strongly acidic conditions (pH = 3.0), the removal efficiency decreased, which can be explained by the scavenging of sulfate radicals (SO_4_^•-^) and hydroxyl radicals (^•^OH) by excess H^+^ ions, which suppresses the overall oxidative activity of the system (Jing et al., 2024). With the variation of pH value for alkaline conditions, the degradation efficiency remained consistently high. The performance is attributed to the PMS self–decomposition and the predominance of HSO_5_^−^, which interacts favorably with the catalyst surface (Luo et al., 2025). Considering the superior and stable activity of Cu_2_Zn_1_-MOFs/PMS system in the pH range of 5.0–9.0, which is highly representative of natural water conditions, further experiments was carried out using CBZ solutions at original pH without additional adjustment.

In actual water matrices, inorganic anions and natural organic matter (NOM) play a key role on the catalytic activation of peroxymonosulfate (PMS) and the consequent degradation of organic pollutants (Zhang et al., 2024). To evaluate the stability and applicability of Cu_2_Zn_1_-MOFs/PMS system in environmentally aqueous conditions, the effects of common inorganic ions (Cl^−^, SO_4_^2-^, CO_3_^2-^, and NO_3_^−^; each at 10 mM) on CBZ degradation were investigated. As shown in Figure 2f, after 15 min, the CBZ removal efficiencies in the presence of Cl^−^, SO_4_^2-^, CO_3_^2-^, and NO_3_^−^ were 90.5%, 76.5%, 81.9%, and 86.9%, respectively. The corresponding pseudo–first–order rate constants were 0.1479 min^-1^, 0.0705 min^-1^, 0.0839 min^-1^, and 0.1099 min^-1^, respectively. Compared to the control experiment, the presence of 10 mM Cl^−^ resulted in a slight decrease of the degradation rate, which can be attributed to the formation of other chlorine species (e.g., Cl^•^ and Cl_2_^•-^) through the reactions with PMS derived radicals (Equations 4–6) (Fang et al., 2022; Xu et al., 2025). Similarly, the addition of SO_4_^2-^ exhibited the weakest degradation efficiency within 15 min due to the scavenging effect of SO_4_^•-^ radicals, thereby reducing the available active radicals for CBZ degradation (Equation 7) (Dong et al., 2025). CO_3_^2-^ and NO_3_^−^ ions exhibited a mild inhibitory effect which is ascribed to the conversion of highly reactive ^•^OH into less reactive carbonate radicals (CO_3_^•-^) and (NO_3_^•-^) (Equations 8, 9) (Yang et al., 2024). To further assess the impact of natural organic matter, humic acid (HA) was selected as a representative NOM. As depicted in Supplementary Figure S8, HA at concentrations of 10 and 30 mg L^-1^ can significantly alter the degradation efficiency of CBZ, exerting strong influence on the CBZ removal efficiency and rate constant.

$$Cl^{-}+SO_{4}{\text{​}}^{•-}\to SO_{4}{\text{​}}^{2-}+Cl^{•}$$ (4)

Cl−+O•H→OH−+Cl• (5)

$$Cl^{-}+Cl^{-}\to Cl_{2}{\text{​}}^{•-}$$ (6)

$$SO_{4}{\text{​}}^{2-}+SO_{4}{\text{​}}^{•-}\to S_{2}O_{8}{\text{​}}^{2-}+e^{-}$$ (7)

$$CO_{3}{\text{​}}^{2-}+OH\to CO_{3}{\text{​}}^{•-}+OH^{-}$$ (8)

O•H+NO3​−→NO3​•+OH− (9)

### 3.3 Reusability and stability of Cu_2_Zn_1_-MOFs

**FIGURE 3:**
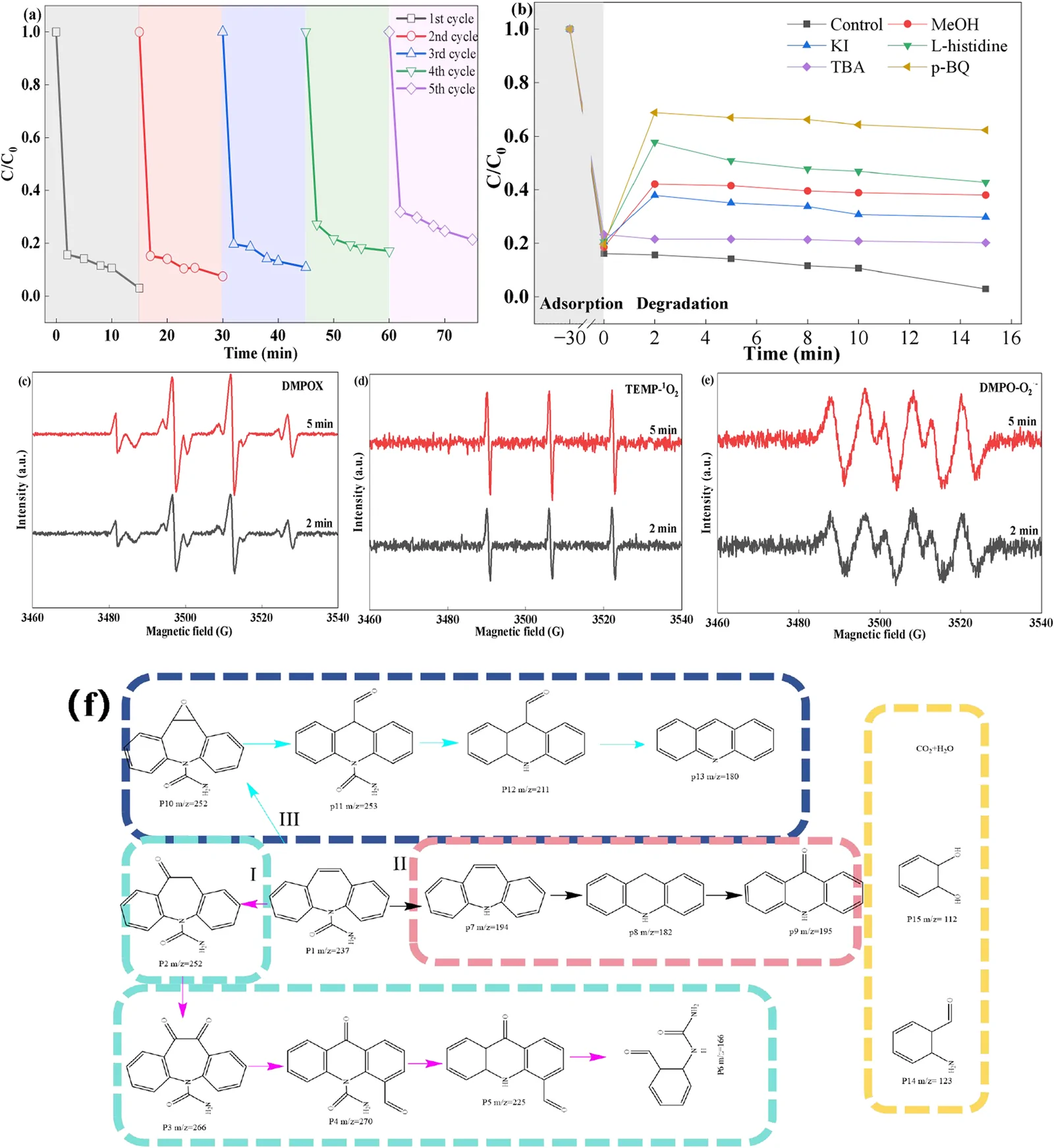
**(a)** Cycle experiments in Cu2Zn₁-MOFs/PMS process; **(b)** quenching experiments on the degradation of Cu2Zn1-MOFS/PMS process. **(c, e)** EPR spectra with DMPO and **(d)** TEMP in the Cu2Znı-MOFs/PMS process, **(f)** CBZ possible degradation pathway in CuxZny-MOFs/PMS process. Conditions: CBZ = 5.0 mg L-¹; catalysts = 0.05 g L-¹; PMS = 0.5 mM; pH = 5.8 (unadjusted); MeOH = TBA = 500 mM; KI = 10 mM; L-histidine = 1.0 mM; p-BQ = 10 mM.

Reusability and sustained catalytic stability are essential indicators for evaluating the practical viability of heterogeneous catalysts. Accordingly, the recycling performance of Cu_2_Zn_1_-MOFs were systematically examined. As illustrated in Figure 3a, catalytic efficiency in the Cu_2_Zn_1_-MOFs/PMS showed gradually decreased over four consecutive cycles. The adsorption and degradation performance of the catalyst decreased after cycling, mainly due to the adsorption and accumulation of organic intermediates on the active sites of catalyst, resulting in the reduction of ROS generation (Du et al., 2026; Thai et al., 2024; Zou et al., 2026). In addition, the metal ions would gradually be leaching from the catalyst framework, where the Cu and Zn ions leaching during the catalytic process was quantified by ICP–OES technology. The concentration of leached Cu ions tested below 1.31 mg·L^-1^, while Zn leaching was under 1.08 mg·L^-1^. Although these values are very close to the standards for drinking water quality in China ([Cu]<1.0 mg·L^-1^, [Zn]<1.0 mg·L^-1^), copper and zinc ions are recognized as potentially hazardous, highlighting the critical urge to limit their discharge and release.

Supplementary Figure S6 illustrates the XRD patterns of the fresh and used Cu_2_Zn_1_-MOFs catalyst in the degradation reaction. A comparison of XRD patterns demonstrates that the characteristic diffraction peaks of Cu_2_Zn_1_-MOFs remain distinguishable after PMS activation, suggesting that the primary overall crystal structure is maintained. Some slight differences in peak intensity are observed which are attributed to deposition of organic degradation intermediates or minor surface oxidation of metal sites on the catalyst surface. In addition, the typical diffraction signals assigned to monometallic metal oxides was much lower in the spent catalyst. Furthermore, SEM images (Supplementary Figure S7) verifies that the morphology of the catalyst is well preserved after the reaction. The catalyst still maintains an aggregated structure assembled by two–dimension nanoparticles, with no obvious sintering phenomenon and structural collapse. Despite the slight change in the surface functional groups during the catalytic reaction, these above characterization results demonstrate that the catalyst framework and morphology of Cu_2_Zn_1_-MOFs catalyst are well retained, explaining the good catalytic performance in the first three cycles.

### 3.4 Possible catalytic mechanisms for CBZ removal in the Cu_2_Zn_1_-MOFs/PMS process

Radical scavenging experiments were carried out to determine the dominant reactive oxygen species produced in the Cu_2_Zn_1_-MOFs/PMS system. Without the addition of any quencher, the catalytic efficiency achieved 97.83% CBZ removal within 15 min. Systematic evaluation was performed by using different radical scavengers to quantify the relative contributions of reactive species during CBZ degradation. To eliminate the interference of competitive adsorption reaction between quenchers and CBZ, all quenching experiments were conducted after reached the adsorption–desorption equilibrium to identify the dominant reactive species in CBZ degradation (Figure 3b). Since adsorption process constitutes an essential step in the removal systems, the presence of competing quenchers could compete for surface active sites and modify the surface properties, resulting in the reduced removal efficiency. In general, to evaluate the ROS contributes to the CBZ degradation, MeOH is used to quench hydroxyl radicals (^•^OH, k = 2.5 × 10^7^ M^-1^ s^-1^) and sulfate radicals (SO_4_^•-^, k = 9.7 × 10^8^ M^-1^ s^-1^), TBA to quench ^•^OH, p-BQ to quench O_2_^•-^, KI to surface–bound radicals, L-histidine to quench ^1^O_2_ (Ji et al., 2024; Zhou et al., 2026). As demonstrated in Figure 3b, with the introduction of 500 mM TBA and MeOH as radical scavengers, CBZ removal reduced to 79.77% and 61.95%, respectively. These results indicated that the addition of TBA partially inhibited the degradation process, whereas MeOH exhibited stronger suppression, implying disparate quenching effects on the radical mediated–oxidation pathways. With the addition of potassium iodide (KI, 10 mM), the removal of CBZ was 70.28%, which can be ascribed to the surface–bound radical quenching effect. Moreover, in the presence of L-histidine (L-his, 1 mM), the CBZ removal efficiency declined to 57.19%, confirming the generation of singlet oxygen in the reaction due to the high reactivity with electrophilic ^1^O_2_. Additionally, CBZ degradation efficiency was suppressed with the presence of furfuryl alcohol (0.1 mM and 0.2 mM), where 0.2 mM furfuryl alcohol showed 23.9% CBZ removal, as shown in Supplementary Figure S8. These results indicated that singlet oxygen (^1^O_2_) was generated, implying non–radical pathway occurred in the PMS activation process. Moreover, it was considered that the formation of singlet oxygen was along with the production of superoxide radicals (O_2_^•-^). When 10 mM p-BQ was added, the CBZ removal efficiency decreased to 37.69%, suggesting that the O_2_^•-^ radicals was also participate in the degradation process and served as an important reactive species during the degradation process.

Furthermore, electron paramagnetic resonance (EPR) spectroscopy was performed to directly identify the reactive oxygen species (ROS) generated in the Cu_2_Zn_1_-MOFs/PMS system. 5, 5-Dimethyl-1-pyrroline N-oxide (DMPO) was used as a spin–trapping capture for sulfate radicals (SO_4_^•-^), hydroxyl radicals (^•^OH), and superoxide radicals (O_2_^•-^), while 2, 2, 6, 6-tetramethyl-4-piperidine (TEMP) was used for singlet oxygen (^1^O_2_) (Chen et al., 2025). As shown in Figure 3c, the peak signals of DMPO–X were observed, attributing to the reaction between DMPO and sulfate radicals and hydroxyl radicals. The peak can be assumed by the characteristic peak of DMPO–X (X presented for SO_4_^•-^ or ^•^OH), evidencing the production of reactive radicals. The characteristic peak of singlet oxygen (TEMP–^1^O_2_) was detected, revealing the prompt activation of PMS and subsequent formation of singlet oxygen in Cu_2_Zn_1_-MOFs/PMS process (Figure 3d). Based on the above results, the quenching experiment and EPR analyses jointly demonstrate that CBZ degradation process underwent both radical and non–radical pathways. Sulfate radicals (*in situ* generated hydroxyl radicals), superoxide radical and singlet oxygen are all recognized as the main reactive species for the oxidative CBZ degradation process.

### 3.5 Identification of active sites and activation of Cu_2_Zn_1_-MOFs/PMS process

**FIGURE 4:**
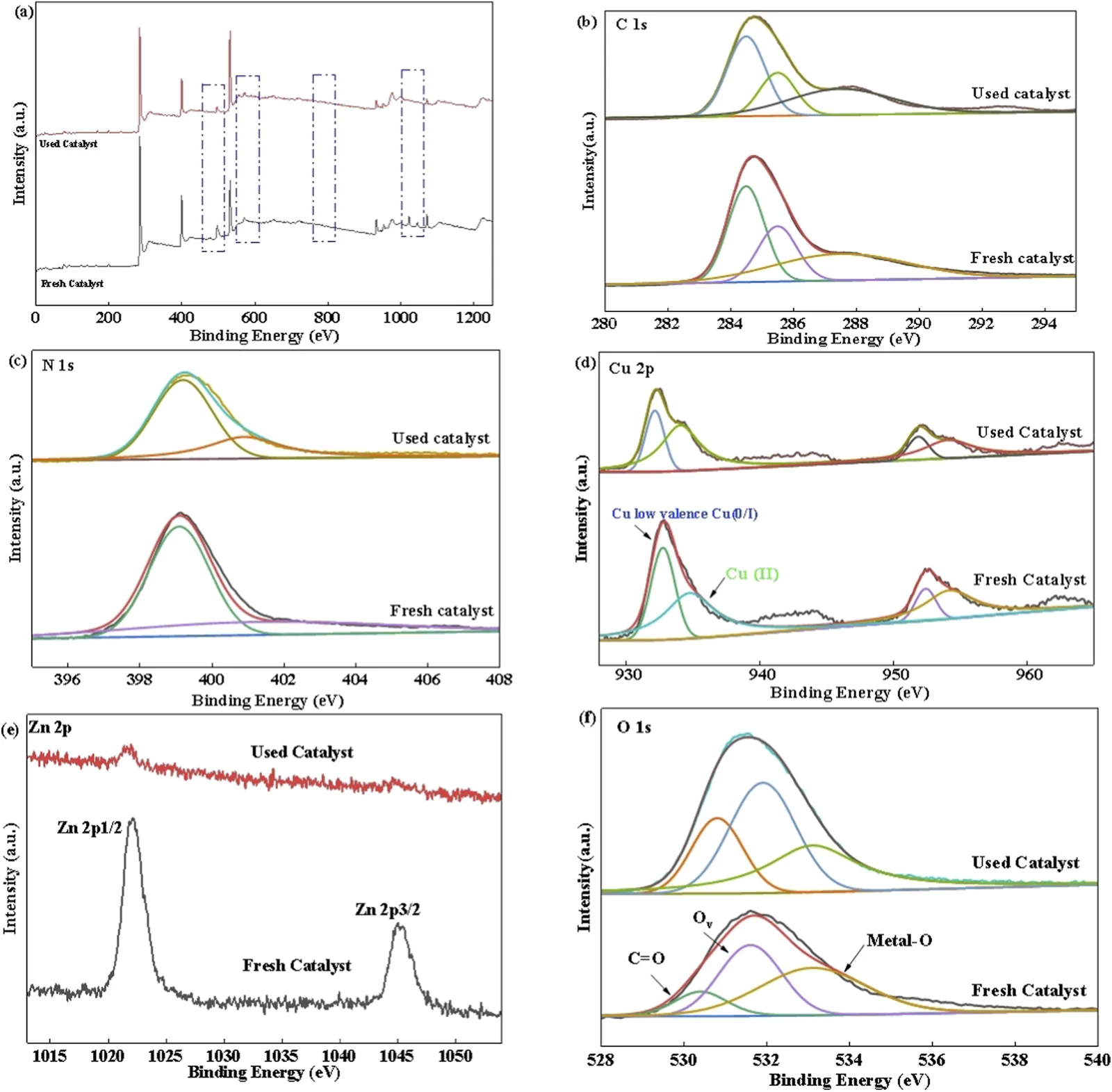
High-resolution XPS spectra of Cu2Zni-MOFs catalyst before and after reaction. **(a)** XPS survey, **(b)** C 1s, **(c)** N 1s, **(d)** Cu 2p, **(e)** Zn 2p, **(f)** O 1s.

XPS analysis was employed to identify the elemental composition and chemical valence states of the catalyst before and after the PMS reaction. As presented in Figure 4a, the XPS spectra confirmed that Cu_2_Zn_1_-MOFs was composed of C, N, O, Cu, and Zn elements. The high–resolution C1s spectrum (Figure 4b) was deconvoluted into three characteristic peaks at 284.5 eV, 285.5 eV and 287.5 eV, which were assigned to C–C, C–N, and C=N bonds, respectively. There are no substantial changes after the reaction, suggesting that the carbon species underwent no significant variation. The N 1s spectrum (Figure 4c) exhibited two distinct peaks at 399.1 eV and 401.1 eV, corresponding to C–N and N–H species. Furthermore, analysis of the N 1s region (Figure 4c) indicated a decrease from 72.2% to 66.1% in N–N bonds after the reaction. Figure 4d displays the Cu 2p spectrum, which could be fitted into mixed Cu(II) and Cu(I/0) valence states. The characteristic peaks at 934.7 eV and 952.4 eV were assigned to Cu(II), whereas at 932.7 eV and 954.4 eV corresponded to Cu(I/0), indicating that copper exhibits multi–valency coexistence within the catalyst (Yin et al., 2025). In the Cu 2p species, the contents of Cu(II) decreased from 69.7% to 59.6%, while Cu(I/0) increased from 30.3% to 41.3%. The results indicated that electron transfer from high valence to low valence was occurred on the catalyst surface, while the redox potentials of Cu and Zn pairs facilitates the catalytic activity (Han et al., 2025). As for the Zn 2p spectrum (Figure 4e), two characteristic peaks at binding energies of ∼1,022.2 eV (Zn 2p3/2) and ∼1,045.2 eV (Zn 2p1/2) are displayed, with a spin–orbit splitting energy of 23 eV. These peaks are characteristic of Zn, indicated that the Zn^2+^ on the catalyst surface was stable within the MOF framework in the Zn(II) oxidation state. No metallic Zn^0^ peaks were detected, indicating that Zn maintains its divalent state throughout the coordination process. The presence of O element in the bimetallic Cu_2_Zn_1_-MOFs, which were centered at 530.5 eV, 531.6 eV and 533.1 eV corresponding to C=O, O_v_ and metal–O, could be ascribed to residual oxygen–containing species, such as adsorbed oxygen or residual organic solvents, remaining in the catalyst (Figure 4f). The O 1s spectra showed a decline in oxygen vacancy (O_v_) after the reaction, confirming that the direct participation of O_v_ active sites in the process. Such oxygen vacancies are prone to facilitate the adsorption of PMS on the catalyst surface and further contribute to the production of superoxide radical and singlet oxygen (Fang et al., 2023). The presence of oxygen vacancies endows MOFs with abundant structural imperfections, which greatly facilitates interface charge migration and further optimizes the catalytic reaction activity. The above XPS results verified the successful synthesis of Cu_2_Zn_1_-MOFs. With the introduction of bimetallic metals, the electron transfer was accelerated which could facilitate the PMS activation process.

**FIGURE 5:**
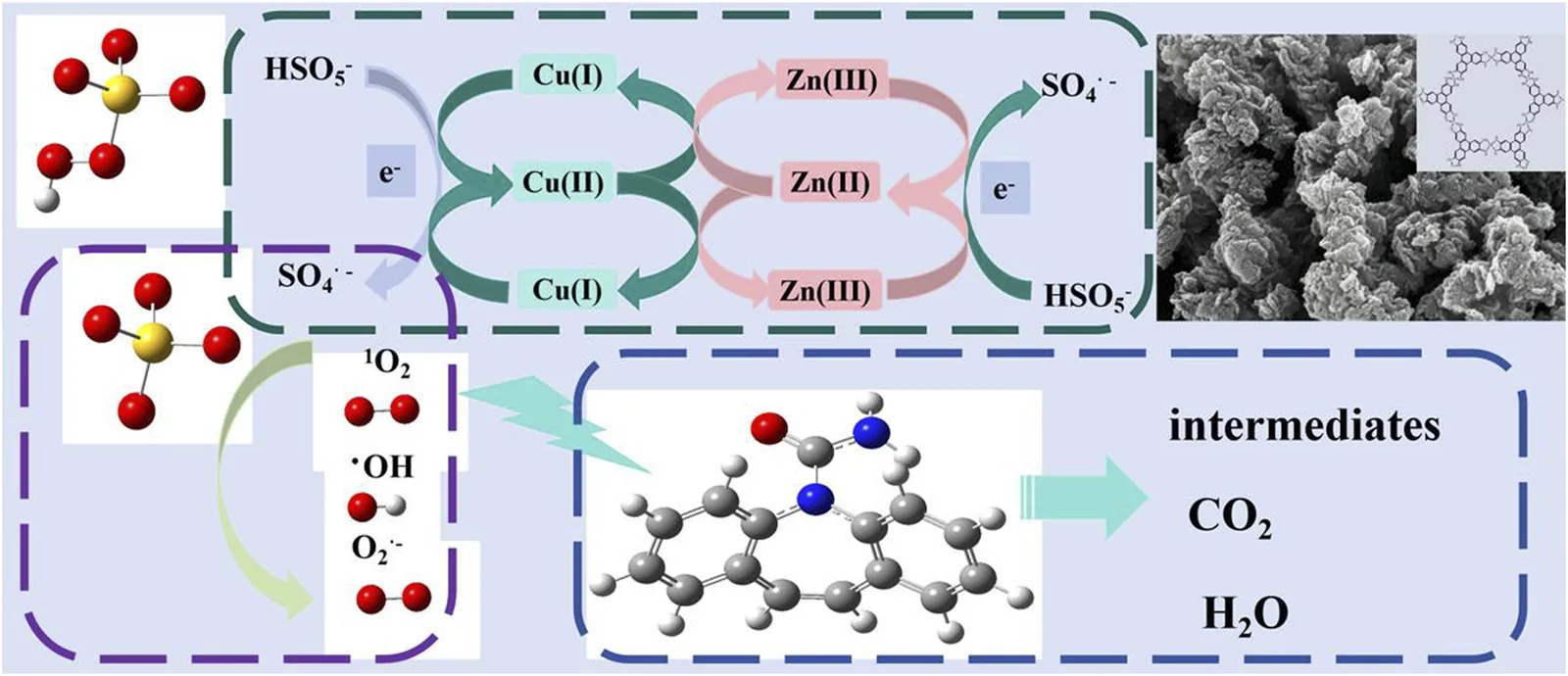
Schematic catalytic mechanism of Cu2Zn1-MOFs catalyst in PMS activation.

Furthermore, the Cu_x_Zn_y_-MOFs/PMS catalytic system exhibited a limited mineralization performance for CBZ, achieving a total organic carbon (TOC) removal efficiency reaching approximately 14.3% (Supplementary Figure S9). This observation TOC results imply that most intermediate degradation products were not completely converted into inorganic small molecules in the current reaction conditions. To further assess the elimination performance of CBZ in practical aqueous matrices, degradation experiments were conducted using different water matrix, as summarized in Supplementary Figure S10. Experimental results reveal that the optimal CBZ degradation efficiency was achieved in deionized water, whereas markedly reduced efficiencies were observed in tap water (86.38%), mineral water (57.91%), and rain water (37.53%). Therefore, compared with deionized water, the CBZ degradation efficiency was inhibited in the other three kinds of water matrix. The inorganic ions, chromaticity color and dissolved organic substance could affect the CBZ degradation efficiency. Generally, the Cu_2_Zn_1_-MOFs/PMS system exhibits considerable potential for industrial wastewater remediation, coexisting ions in natural water severely affect its catalytic activity. Nevertheless, further efforts should focus on boosting its degradation performance to realize practical treatment through this advanced oxidation process. On the basis of quenching experiments, EPR analysis and XPS analysis, the catalytic mechanism of PMS activation to generate reactive oxygen species was summarized, as shown in Figure 5.

The low–valence copper and zinc sites on Cu_2_Zn_1_-MOFs can activate the peroxymonosulfate (HSO_5_^−^) to generate SO_4_^•-^ and ^•^OH, while can also react with dissolved O_2_ to form O_2_^•-^ and singlet oxygen (^1^O_2_). In the meanwhile, the high–valence copper and zinc sites can also react with HSO_5_^−^ and SO_5_^•-^ was produced. These processes sustain a continuous redox cycle between copper and zinc, as represented in Equations 10–12. The standard reduction potential of zinc was lower than copper, Zn pair can promote the regeneration of copper, enhancing the degradation efficiency. Furthermore, the generated SO_4_^•-^ can be partially converted to ^•^OH through the reaction with OH^−^ or water, as shown in Equation 13 (Zhu et al., 2025). The XPS results also indicate that ^1^O_2_ can be generated through the transformation of O_2_^•-^ by oxygen vacancy (O_v_) mediated pathways and PMS self–decomposition process, described in Equations 14–17 (Meng et al., 2026). Fundamentally, the CBZ molecule is attacked by these reactive oxygen species, leading to its oxidation into intermediate products or complete mineralization to CO_2_ and H_2_O.

$$Catalyst-Cu\left(I\right)+HSO_{5}{\text{​}}^{-}\to Catalyst-Cu\left(II\right)+SO_{4}{\text{​}}^{•-}+OH^{-}$$ (10)

$$Catalyst-Cu\left(II\right)+HSO_{5}{\text{​}}^{-}\to Catalyst-Cu\left(I\right)+SO_{5}{\text{​}}^{•-}+H^{+}$$ (11)

$$HSO_{5}{\text{​}}^{-}\to SO_{5}{\text{​}}^{•-}+H^{+}+e^{-}$$ (12)

2SO4​•−+H2O→O•H+H++SO4​2− (13)

$$O_{V}+e^{-}\to O_{2}{\text{​}}^{•-}$$ (14)

$$O_{V}+HSO_{5}{\text{​}}^{-}\to O_{2}{\text{​}}^{•-}+HSO_{4}{\text{​}}^{-}$$ (15)

$$O_{2}{\text{​}}^{•-}+HSO5^{-}\to^{1}O_{2}+HSO_{4}^{-}$$ (16)

SO4​•−+O•H+O2​•−+1O2+CBZ→intermediates→CO2+H2O (17)

### 3.6 DFT calculations and degradation pathways of CBZ

**FIGURE 6:**
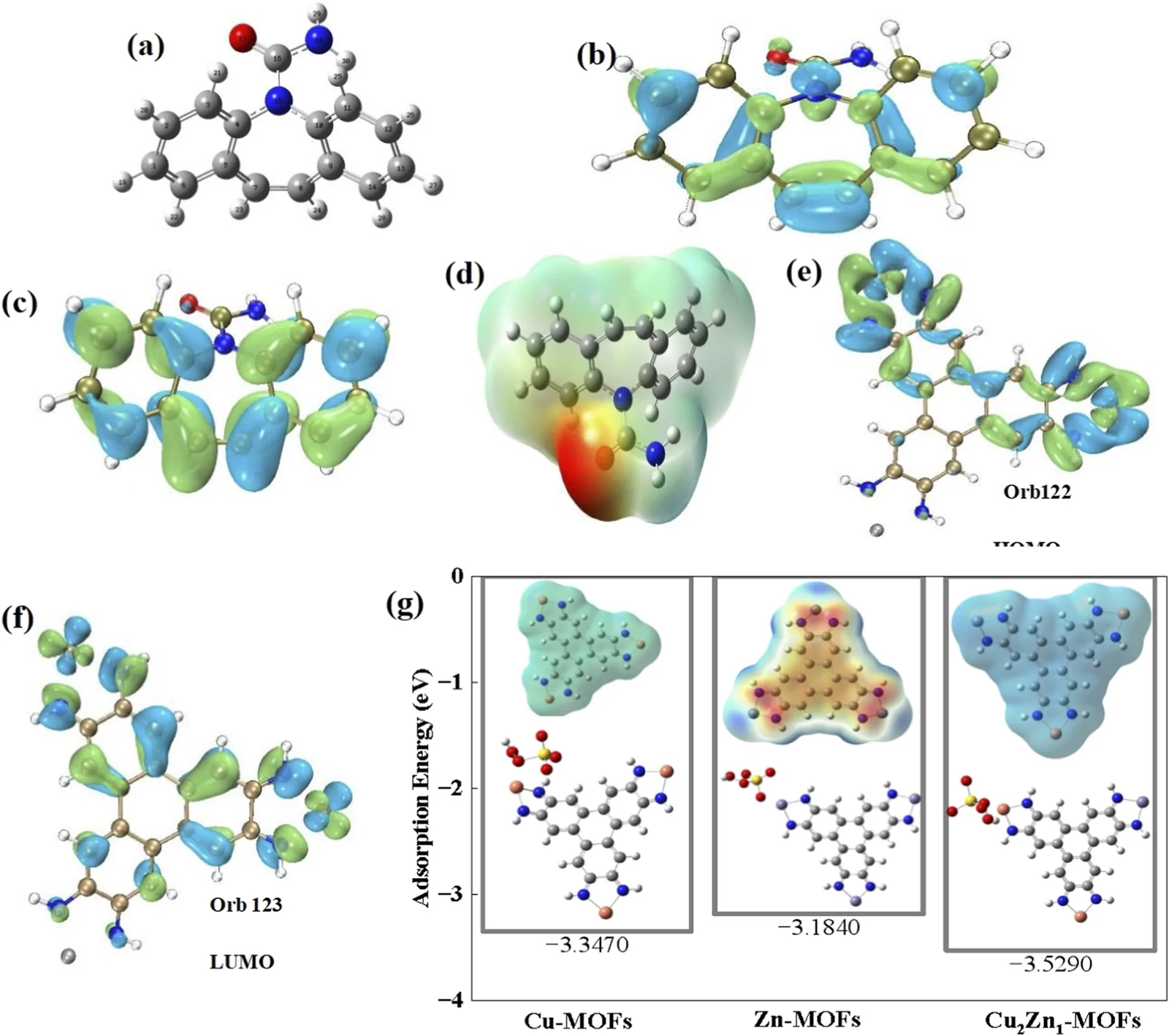
**(a)** Atomic number of CBZ, **(b, c)** LUMO and HOMO, **(d)** Surface electrostatic potential, **(e-f)** optimized HOMO and LUMO of Cu2Zn₁-MOFs, **(g)** adsorption energy of Cu-MOFs, Zn-MOFs and Cu2Zn1-MOFs.

To gain insights into the degradation pathway of carbamazepine (CBZ), theoretical calculations of CBZ molecules were performed by using Gaussian 09W software. As shown in Figures 6a–c, the highest occupied molecular orbital (HOMO) with representing electron–rich regions is primarily localized on the nitrogen atom N1, C7 and C8, as well as the benzene rings symmetrically attached to the central heterocyclic core. The lowest unoccupied molecular orbital (LUMO), indicating electron–deficient sites, is predominantly distributed over C5, C6, C10, and C14, suggesting that these positions are susceptible to accept electrons during oxidative attacks. The electrostatic potential (ESP) distribution of CBZ (Figure 6d) further demonstrates well–defined electrophilic and nucleophilic regions. Negative ESP values observed at C11, O17, and H25 which corresponded to electron–rich regions, which favor the attraction of positively charged species and prone to attack by electrophilic reactive oxygen species (ROS). By contrast, regions exhibiting more positive electrostatic potentials are more susceptible to nucleophilic ROS attack. It can be assumed that C3, C7, C8, and C11 sites are more vulnerable to be attacked during the CBZ degradation process.

The synergistic effect of the bimetallic Cu_x_Zn_y_-MOFs catalysts was verified by density functional theory (DFT) calculations, based on the first principles methods. In Figures 6e,f and Supplementary Figures S11, S12, it can be observed the corresponding HOMO and LUMO molecular orbital of Cu-MOFs, Zn-MOFs and Cu_2_Zn_1_-MOFs and HOMO–LUMO orbitals gap of Cu-MOFs, Zn-MOFs and Cu_2_Zn_1_-MOFs, respectively. In the heterogeneous catalytic process, it is known that the active sites on the catalyst surface could adsorb peroxymonosulfate (PMS) and lead to the cleavage of the O–O bond to generate sulfate radicals (SO_4_^•-^) and hydroxyl radicals (Liu et al., 2024; Sun et al., 2023). The bimetallic synergistic mechanism can be elucidated by evaluating the adsorption energy between the active sites and PMS molecules. As presented in Figure 6g and Supplementary Table S4, the adsorption energy of PMS on Cu_2_Zn_1_-MOFs can be determined to be −3.529 eV, which is substantially more favorable than the values of −3.347 eV and −3.184 eV observed for monometallic Cu-MOFs and Zn-MOFs, respectively. It is revealed that Cu_2_Zn_1_-MOFs displays enhanced PMS adsorption efficiency, thus favoring the subsequent activation and decomposition of PMS molecules. Thus, the bimetallic two–oriented structure of Cu_x_Zn_y_-MOFs catalysts promoted PMS adsorption and stretched the O–O bond, accelerating its cleavage and enabling more efficient PMS activation toward reactive oxygen species. In the Cu_2_Zn_1_-MOFs/PMS process, both the Cu and Zn served as the active site for PMS activation to produce ROS. The cooperation of Zn could not only directly participate in PMS activation, but also facilitate the regeneration of Cu(I/0) from Cu(II) and improved electron transfer between the catalyst and PMS, enhancing the CBZ degradation efficiency.

Based on the above results, the degradation intermediates of CBZ degradation process were identified by LC/MS in the Cu_2_Zn_1_-MOFs/PMS process, as shown in Supplementary Table S5. It can be seen in Figure 6 that atom (N1), atom (C7) and atom (C8) of CBZ were electron–rich regions which were easy to lose electrons by the attack of reactive oxygen species. In this case, three degradation pathways were proposed according to the detected intermediates (Wu Y. et al., 2025). Through pathway I, P2 was yielded due to the attack of the olefinic double bond at the atom (C8) position of the central heterocycle in CBZ by reactive oxygen radicals which was in good agreement with DFT predictions identifying atom (C7) and atom (C8) as vulnerable sites. P2 is further oxidized to form P3, which might be underwent the cleavage of the central heterocycle and oxidation of the hydroxyl group to generate P4. P5 is formed via the cleavage of amido groups in P4, which might be oxidized to form P6. In pathway II, the C–N bond was undergoing cleavage to form compound P7 where is predicted high activity of atom (N1) sites. Due to further hydroxylated and oxidation process, P8 and P9 was generated. In pathway III, the superoxide radical and singlet oxygen generated during the reaction easily oxidized the electron–rich olefinic double bond of CBZ, causing the formation of P10. P10 could further undergo dehydrogenation and amide cleavage to produce P11. In another hand, the cleavage of amido group in P11 could lead to the formation of P12, which could also further oxidize to P13. The subsequent intermediates were undergoing further ROS attack, resulting in bond cleavage and ring–opening occurrence, eventually degraded to low-molecular-weight intermediates (P14, P15) or complete mineralization to CO_2_ and H_2_O.

## 4 Conclusion

A series of Cu_x_Zn_y_-MOFs catalyst was synthesized via a solvothermal approach and employed as a high–performance catalyst for peroxymonosulfate (PMS) to achieve rapid degradation of carbamazepine (CBZ) in 15 min. The catalyst exhibited remarkable superior activity achieved 97.83% CBZ removal. Catalyst characterization was carried out by SEM, TEM/EDS, FTIR, XRD and XPS revealed that the synergistic of bimetallic Cu–Zn groups and oxygen vacancies (O_v_) served as active sites responsible for the enhanced catalytic performance. Optimal catalytic efficiency was attained with a Cu/Zn atomic ratio of 2:1. After introducing bimetallic metal ions to MOFs–derived structure, the specific surface area, pore size and pore volume was increased, which benefit the followed oxidation process with more active site participated. Such a synergistic effect improved the adsorption capacity and degradation performance, realizing the integration of adsorption and degradation. The degradation rate of Cu_2_Zn_1_-MOFs (0.1833 min^-1^) was higher than monometallic Cu-MOFs and Zn-MOFs. Quenching experiments and EPR analysis identified sulfate radicals (SO_4_^•-^), hydroxyl radical (^•^OH), superoxide radicals (O_2_^•-^), and singlet oxygen (^1^O_2_) as the dominant reactive species during the CBZ oxidation process. Further theoretical calculation with LC/MS analysis, plausible degradation pathways for CBZ were demonstrated, providing insight into the oxidative transformation process. This study not only presents an efficient PMS activator but also expands the application scope of MOFs–derived catalyst in sulfate radical–based advanced oxidation processes.

## Data Availability

The original contributions presented in the study are included in the article/Supplementary Material, further inquiries can be directed to the corresponding author.
