# Supplementary material for: Synthesis and applications of bimetallic metal–organic frameworks catalyst for activating peroxymonosulfate for carbamazepine degradation
Source: Front Chem. 2026 Aug 28;14:1891127. doi: 10.3389/fchem.2026.1891127 (PMC13561988; doi:10.3389/fchem.2026.1891127)
Supplement: Supplementary file 1 [file DataSheet1.docx]

Supplementary Material

**Synthesis and applications of bimetallic metal**–**organic frameworks catalyst for activating peroxymonosulfate for carbamazepine degradation**

Xin Zhong^1, *^, Qi Huang^2^, Meihuan Ji^1^, Zhao Liao^2^, Chaosheng Chen^2^

*^1^ Experimental Teaching Platform, Beijing Normal University at Zhuhai, Zhuhai 519000, PR China;*

*^2^ Faculty of Arts and Sciences, Beijing Normal University, Zhuhai 519000, PR China.*

**^*^Corresponding Author: Xin Zhong** E-mail address: [zhongxin@bnu.edu.cn](mailto:zhongxin@bnu.edu.cn)

# 1 Supplementary Texts

**Texts Lists**

**Text S1** Reagents and chemicals

**Text S2** Catalyst synthesis procedure

**Text S3** Catalyst characterization

**Text S4** Experimental Procedures

**Text S1 Reagents and chemicals**

Copper nitrate hexahydrate (Cu(NO_3_)_2_·3H_2_O, AR), Zinc nitrate (Zn(NO_3_)_2_·6H_2_O , AR), peroxymonosulfate (2KHSO_5_·KHSO_4_·K_2_SO_4_, Oxone@, AR), N, N-Dimethylformamide (DMF, AR), and methanol (MeOH, AR) were purchased from Shanghai McLean biological Co., Ltd. Tert-butyl alcohol (TBA, >99%, AR), 1, 4-Benzoquinone (p-BQ, AR) and methanol (HPLC grade) were purchased from Xilong Scientific Co., Ltd. Sodium nitrate (NaNO_3_, AR), sodium chloride (NaCl, AR), sodium sulfate (Na_2_SO_4_, AR), sodium carbonate (Na_2_CO_3_, AR), and sodium hydroxide (NaOH, AR) were supplied by Sinopharm Chemical Reagent Co., Ltd. Carbamazepine (CBZ, >98%, AR) and 2,3,6,7,10,11-hexaaminotriphenylene (HATP·6HCl, >98%) were provided by Shanghai McLean biological Co., Ltd. All the chemical reagent was used as received without further purification.

**Text S2 Catalyst synthesis procedure**

The series of Cu_x_Zn_y_-MOFs catalyst was synthesized using a solvothermal method. In general, Cu(NO_3_)_2_·3H_2_O and Zn(NO_3_)_2_·6H_2_O were used as the metal source of copper and zinc for the subsequent synthesis, respectively. At the beginning, 0.15 mmol of mixed metal salts with different molar ratio of Cu to Zn (3:1, 2:1, 1:1, 1:2 and 1:3), were added to 30 mL DMF under vigorous stirring as solution I. Then, 0.15 mmol HATP·6HCl was dissolved in 10 ml water as solution II and slowly introduced dropwise to solution I. 1 mL NaOH aqueous solution (6.0 mol·L^-1^) was added in the mixed solution, and stirred at 65 °C for 180 min. The resulting powder was centrifuged and washed with water and methanol and vacuum dried at 60 °C overnight. The synthesized Cu_x_Zn_y_-MOFs was named according to the initial copper to zinc dosage ratio. At the same time, Cu-MOFs and Zn-MOFs was also synthesized by adding single metal source.

**Text S3** **Catalyst characterization**

The characterization of the Cu_x_Zn_y_-MOFs catalysts was carried out by using X–ray powder diffraction (XRD, XRD–7000, SHIMADZU, Tokyo, Japan), X–ray photoelectron spectroscopy (XPS, Nexsa G2, Thermo Fisher, Waltham, MA, USA), and Fourier–transform infrared spectroscopy (FT-IR, Nicolet iS5, Thermo Fisher, USA). The morphologies were investigated using scanning electron microscopy (SEM, Zeptools, Hefei, China) and transmission electron microscopy (TEM, JEM F200, Tokyo, Japan), and the distributions of different elements were directly analyzed by energy dispersive spectrometer (EDS). The EPR test was performed to detect the reactive oxygen species on a Bruker X–band spectrometer with DMPO and TEMP as the spin–trapping agents. The N_2_ adsorption–desorption isotherms and pore size distribution are studied by Brunauer–Emmett–Teller (BET) analysis using BELSORP MAX X. The inductively coupled plasma (ICP) analysis was performed using the (ICP/OES) instrument to measure the leaching of metal ions in the liquid after the reaction (iCAP 7200 Duo, Thermo Fisher, USA).

The TOC removal was determined by vario TOC analyzer (Elementar, German). The degradation intermediates were identified using LC–MS/MS (Waters Q–tof, Milford, MA, USA), and a proposed degradation pathway was given. The radical quenching experiments were carried out to trap the free radicals, such as MeOH (sulfate radical and hydroxyl radical), TBA (hydroxyl radical), L-histidine and furfuryl alcohol (singlet oxygen), p-BQ (superoxide radical).

**Text S4** **Experimental Procedures**

In this study, carbamazepine (CBZ) was selected as the target pollutant to evaluate the catalytic performance of Cu_x_Zn_y_-MOFs catalyst in the PMS activation. The catalytic activity, stability, and reusability were systematically evaluated via batch experiments under different experiment conditions, including initial pH, catalyst dosage, PMS concentration, coexisting ion concentrations and nature organic matter (Humid Acid, HA). At the beginning of the typical experiment, 0.05 g·L^-1^ catalyst was added to the CBZ solution with an initial CBZ concentration of 5.0 mg·L^-1^ (pH = 5.8; the solution volume was 100 mL). The mixed solution was stirred at 25 °C for 30 min for adsorption, followed by the dispersion of 0.5 mM PMS in the solution. In the quenching experiments, the mixed solution was first to reach adsorption–desorption equilibrium, then the quenchers were added in the reaction to eliminate the possible interference arising from the quenchers competing for adsorption sites.

At a predetermined time–interval, 1 ml of the reaction solution was extracted for analyzing. The concentration of CBZ was detected by high performance liquid chromatography (Shimadzu HPLC/LC–16, C18 column, 4.6×250 mm, 5 µm). The injection volume of HPLC was 20 μL and methanol and ultra–pure aqueous solution (70:30, v/v) were used as the mobile phase. The flow rate was set at 1.0 mL·min^-1^ and the column temperature was maintained at 25 ℃. The UV detection wavelength was set to 286 nm for CBZ.

The degradation efficiency of CBZ in the Cu_x_Zn_y_-MOFs/PMS system was fitted using a first–order reaction kinetics equation, as shown in Equation (1):

$\ln(C_{t}/C_{0})=-k_{obs}t$ (1)

where C_t_ and C_0_ are the CBZ concentrations (mg·L^-1^) at reaction time t and the initial time, respectively; t is the reaction time (min); and k_obs_ is the pseudo–first–order kinetic constant (min^-1^).

Two models were used for Cu_x_Zn_y_-MOFs, i.e., pseudo–first–order dynamic model (Eq. 2) and pseudo–second–order dynamic model (Eq. 3) to carry out fitting the adsorption equilibrium data of CBZ. The expression is as follows:

$$\ln\left( \text{q}_{\text{e}}\text{-}\text{q}_{\text{t}} \right)\text{=}\text{ln}\text{q}_{\text{e}}\text{-}\text{k}_{\text{1}}\text{t}$$

(2)

$$\frac{\text{t}}{\text{q}_{\text{t}}}\text{=}\frac{\text{1}}{\text{k}_{\text{2}}\text{q}_{\text{e}}^{\text{2}}}\text{+}\frac{\text{t}}{\text{q}_{\text{e}}}$$

(3)

Where *q_e_* and *q_t_* are the adsorption capacity of CBZ (mg·g^-1^) at equilibrium and time *t*, respectively, *k_1_* (min^−1^) and *k_2_* (g·mg^−1^·min) are the rate constants.

To elucidate the degradation pathway and reaction sites of CBZ molecules and the adsorption energy of catalyst, first-principles calculations were performed via density functional theory (DFT) implemented in the Gaussian 09W program. All structures were optimized and characterized by 6-31G (d, p) for all the atoms. Selected computed structures and chemical molecules are illustrated using the Multiwfn 6.4 software, Gaussian view 05 software and VMD program [1, 2]. The chemical molecules are presented and drawn using Gaussian view 05 software and ChemOffice software.

[1] Tian Lu, Feiwu Chen, Multiwfn: A Multifunctional Wavefunction Analyzer, J. Comput. Chem. 33, 580-592 (2012) DOI: 10.1002/jcc.22885

[2] Tian Lu, A comprehensive electron wavefunction analysis toolbox for chemists, Multiwfn, J. Chem. Phys., 161, 082503 (2024) DOI: 10.1063/5.0216272

# 2 Supplementary Figures and Tables

## 2.1  Supplementary Tables

## Tables Lists

**Table S1** BET, total pore volume and pore diameter of Cu-MOFs, Zn-MOFs and Cu_2_Zn_1_-MOFs.

**Table S2** Comparison of Cu-based and Zn-based MOFs for pollutants degradation.

**Table S3** The metal leaching concentration of Cu_2_Zn_1_-MOFs catalysts.

**Table S4.** The adsorption energy of Cu_x_Zn_y_-MOFs for PMS.

**Table S5.** List of CBZ main intermediates formed during the Cu_2_Zn_1_-MOFs/PMS degradation.

**Table S1** BET, total pore volume and pore diameter of Cu-MOFs, Zn-MOFs, and Cu_2_Zn_1_-MOFs.

| Materials | S_BET_ (m^2^/g) | PV_Total_ (cm^3^/g) | Pore diameter (nm) |
| --- | --- | --- | --- |
| Cu-MOFs | 92.203 | 0.2812 | 7.089 |
| Zn-MOFs | 8.5909 | 0.04676 | 1.6314 |
| Cu_2_Zn_1_-MOFs | 157.17 | 0.2977 | 7.3226 |

**Table S2** Comparison of Cu-based and Zn-based MOFs for pollutants degradation.

| Catalysts | Concentration of target pollutants (mg·L^-1^) | Efficiency | Time | Catalyst Dosage | PMS Concentration | Ref |
| --- | --- | --- | --- | --- | --- | --- |
| Cu-MOF | Bisphenol A, 25 mg·L^-1^ | 98% | 30 | 1.0 g·L^-1^ | 1.5 g·L^-1^ | [1] |
| Cu-Fe-MOF | phenol, bisphenol A, 2,4-dichlorophenol, methyl blue, rhodamine B, tetracycline, sulfamethoxazole, 20 mg·L^-1^ | Over 95% | 30 | 200 mg·L^-1^ | 3 mM | [2] |
| CuCo-MOF | p-nitrophenol,  5 mg·L^-1^ | 100% | 30 | 30 mg·L^-1^ | 1.2 mM | [3] |
| Zn-MOF-2.5 | Oxytetracycline, 20 mg·L^-1^ | 93.01% | 90 | 400 mg·L^-1^ | 200 mg/L | [4] |
| CoCu-S | Norfloxacin,  5 mg·L^-1^ | 97.94% | 20 | 50 mg·L^-1^ | 1 mM | [5] |
| Zn/Co MOF | Acetamiprid,  50 mg·L^-1^ | 96.93% | 90 | 10 mg·L^-1^ | 0.1 mM | [6] |
| Co-Cu bimetallic MOF porous carbon | Chloramphenicol, 20 mg·L^-1^ | 98% | 60 | 100 mg·L^-1^ | 1.0 mM | [7] |
| Cu_2_Zn_1_-MOFs | 5 mg·L^-1^ | 97.40% | 15 | 50 mg·L^-1^ | 0.5 mM | This work |

**Reference**

[1] Guan, Y., Zhou, G., Li, L., Jiang, Y., Yin, J., Liu, C., Zhang, L., Han, Q., Ang, E. H. Cu-doped oxygen-rich vacancy MOFs derived perovskite for enhanced mineralization of refractory organics through synergistic non-radical species effects. Separation and Purification Technology, 335, 2024, 126072.
[2] Wang, H., Dai, Y., Wang, Y., Yin, L. One-pot solvothermal synthesis of Cu-Fe-MOF for efficiently activating peroxymonosulfate to degrade organic pollutants in water: Effect of electron shuttle. Chemosphere, 352, 2024, 141333.

[3] Shi, Q., Hou, Y., Zhu, Q., Hao, Y. MOF-derived CuCo carbon microspheres assembled with nitrogen-doped carbon nanotubes as PMS activator for the efficient degradation of p-nitrophenol. Separation and Purification Technology, 354, 2025, 129107.

[4] Bian, S., Sun, W., Pan, Y., Zhang, M., Wu, G., Huang, Y. Zn-MOF derived from waste plastic bottles activated peroxymonosulfate for the degradation of oxytetracycline: Process mechanism and toxicological analysis. Chemical Engineering Journal, 525, 2025, 170288.

[5] Liu, H., Zhang, J., Zhang, L., Cheng, X., Yang, X., Xing, Z., Li, L., Bai, H. MOF-derived 3D bimetallic sulfide for highly efficient activation of peroxymonosulfate: synergistic radical and non-radical degradation of norfloxacin. Separation and Purification Technology, 394, 2026, 137645.

[6] Zhang, M., Li, J., Wu, L., Liang, T., Liu, J., Wang, L. One-step hydrothermal synthesis of Zn/Co MOF for efficiently activating PMS to degrade organic pollutants in water: The reaction kinetics and mechanism. Separation and Purification Technology, 392, 2026, 137127.

[7] Tang, H., Gu, A., Xu, X., Liu, P., Chen, K., Zhou, Y., Gong, C., Wang, P., Yang, Y. Porous carbon catalyst derived from Co/Cu bimetallic MOFs as PMS activator for efficient degradation of chloramphenicol. Journal of Solid State Chemistry, 358, 2026, 125892.

**Table S3** The metal leaching concentration of Cu_x_Zn_y_-MOFs catalysts.

| Catalyst | Copper ion leaching concentration (μg/L) | Zinc ion leaching concentration (μg/L) |
| --- | --- | --- |
| Cu_2_Zn_1_-MOFs/PMS | 1308.4 | 1085.2 |
| Cu-MOFs | 1714.9 | / |
| Zn-MOFs | / | 1161.8 |

**Table S4.** The adsorption energy of Cu_x_Zn_y_-MOFs for PMS the adsorption energy. (**ΔE=E_b_-E_a_-E_o_**)

|  | **E_a_/E_0_ (a.u.)** | **E_b_(a.u.)** | **‌****ΔE (eV)** |
| --- | --- | --- | --- |
| PMS | -774.868 | **_** | **_** |
| Cu-MOFs | −5942.282 | -6717.027 | -3.347 |
| Zn-MOFs | −6359.218 | -7133.969 | -3.184 |
| Cu_2_Zn_1_-MOFs | −6078.617 | -6853.355 | -3.529 |

**E_a_**: energy density of Cu_x_Zn_y_-MOFs; **E_b_**: energy density of Cu_x_Zn_y_-MOFs with PMS; **ΔE**: absorption energy.

**Table S5.** List of main intermediates formed during Cu_x_Zn_y_-MOFs/PMS degradation process of CBZ.

| By-products | Chemical structure | m/z | Formula | HPLC/MS spectra |
| --- | --- | --- | --- | --- |
| CBZ (P1) |  | 237 | C_15_H_12_N_2_O |  |
| P2 |  | 253 | C_15_H_12_N_2_O_2_ |  |
| P13 |  | 180 | C_13_H_9_N |  |
| P3 |  | 271 | C_15_H_14_N_2_O_3_ |  |
| P5 |  | 230 | C_14_H_13_NO_2_ |  |
| P8 |  | 182 | C_13_H_11_N |  |
| P4 |  | 273 | C_15_H_16_N_2_O_3_ |  |
| P6 |  | 164 | C_8_H_8_N_2_O_2_ |  |
| P7 |  | 194 | C_14_H_11_N |  |
| P9 |  | 196 | C_13_H_9_NO |  |
| P10 |  | 253 | C_15_H_12_N_2_O_2_ |  |
| P11 |  | 255 | C_15_H_14_N_2_O_2_ |  |
| P12 |  | 210 | C_14_H_11_NO |  |
| P14 |  | 150 | C_8_H_7_NO_2_ |  |
| P15 |  | 113 | C_6_H_8_O_2_ |  |

## 2.2 Supplementary Figures

**Figures Lists**

**Fig. S1.** Pore diameter distributions of Cu-MOFs, Zn-MOFs and Cu_2_Zn_1_-MOFs catalysts.

**Fig. S2.** FTIR spectra of Cu_2_Zn_1_-MOFs before and after the reaction.

**Fig. S3**. (a) Adsorption curve of Cu_2_Zn_1_-MOFs catalysts. (b) pseudo–first–order kinetic model of Cu_2_Zn_1_-MOFs. (c) pseudo–second–order kinetic model of Cu_2_Zn_1_-MOFs.

**Fig. S4.** Effect of oxidizing agent in the Cu_2_Zn_1_-MOFs/oxidant system. Conditions: CBZ = 5.0 mg·L^-1^; catalysts = 0.05 g·L^-1^; [Oxidant] = 0.5 mM; pH = 5.8 (unadjusted).

**Fig. S5.** Rate constant of CBZ at different metal ratios in Cu_x_Zn_y_-MOFs/PMS process. Condition: CBZ = 5.0 mg·L^-1^; catalysts = 0.05 g·L^-1^; PMS = 0.5 mM; pH = 5.8 (unadjusted).

**Fig. S6.** The XRD patterns of the fresh and used Cu_2_Zn_1_-MOFs in the degradation reaction.

**Fig. S7.** The SEM images of the used Cu_2_Zn_1_-MOFs catalyst in the degradation reaction.

**Fig. S8.** Effects of (a) HA concentration and (b) furfuryl alcohol on catalytic performance. Condition: CBZ = 5.0 mg·L^-1^; catalysts = 0.05 g·L^-1^; PMS = 0.5 mM; pH = 5.8 (unadjusted).

**Fig. S9.** TOC removal on catalytic performance. Condition: CBZ = 5.0 mg·L^-1^; catalysts = 0.05 g·L^-1^; PMS = 0.5 mM; pH = 5.8 (unadjusted).

**Fig. S10.** Different water matrix of Cu_2_Zn_1_-MOFs for PMS activation. Conditions: CBZ = 5.0 mg·L^-1^; catalysts = 0.05 g·L^-1^; PMS = 0.5 mM; pH = 5.8 (unadjusted).

**Fig. S11.** Optimized HOMO and LUMO of (a–b) Cu-MOFs and (c–d) Zn-MOFs.

**Fig. S12.** HOMO–LUMO orbitals gap of Cu-MOFs, Zn-MOFs and Cu_2_Zn_1_-MOFs.

**Supplementary Figure 1.** Pore diameter distributions of Cu-MOFs, Zn-MOFs and Cu_2_Zn_1_-MOFs catalysts.

**Supplementary Figure 2.** FTIR spectra of Cu_2_Zn_1_-MOFs before and after the reaction.

**Supplementary Figure 3.** (a) Adsorption curve of Cu_2_Zn_1_-MOFs catalysts; (b) pseudo–first-order kinetic model of Cu_2_Zn_1_-MOFs (𝑅^2^=0.6, k_1_=0.2005 min^-1^); (c) pseudo–second-order kinetic model of Cu_2_Zn_1_-MOFs (𝑅^2^=0.71, k_2_=0.1267 mg^-1^·min^-1^).

**Supplementary Figure 4.** Effect of oxidizing agent in the Cu_2_Zn_1_-MOFs/oxidant system. Conditions: CBZ = 5.0 mg·L^-1^; catalysts = 0.05 g·L^-1^; PMS = 0.5 mM; pH = 5.8 (unadjusted).

**Supplementary Figure 5.** Rate constant of CBZ at different metal ratios in Cu_x_Zn_y_-MOFs/PMS process. Condition: CBZ = 5.0 mg·L^-1^; catalysts = 0.05 g·L^-1^; PMS = 0.5 mM; pH = 5.8 (unadjusted).

**Supplementary Figure 6.** The XRD patterns of the fresh and used Cu_2_Zn_1_-MOFs catalyst in the degradation reaction.

**Supplementary Figure 7.** The SEM images of the used Cu_2_Zn_1_-MOFs catalyst in the degradation reaction.

**Supplementary Figure 8.** Effects of (a) HA concentration and (b) furfuryl alcohol on catalytic performance. Condition: CBZ = 5.0 mg·L^-1^; catalysts = 0.05 g·L^-1^; PMS = 0.5 mM; pH = 5.8 (unadjusted).

**Supplementary** **Figure 9.** TOC removal on catalytic performance. Condition: CBZ = 5.0 mg·L^-1^; catalysts = 0.05 g·L^-1^; PMS = 0.5 mM; pH = 5.8 (unadjusted).

**Supplementary Figure 10.** Different water matrix of Cu_2_Zn_1_-MOFs for PMS activation. Conditions: CBZ = 5.0 mg·L^-1^; catalysts = 0.05 g·L^-1^; PMS = 0.5 mM; pH = 5.8 (unadjusted).

**(a)**

**Orb124**

**HOMO**

**(b)**

**Orb 125**

**LUMO**

**Orb 126**

**HOMO**

**(c)**

**Orb126**

**HOMO**

**(d)**

**Orb127**

**LUMO**

**Supplementary Figure 11.** Optimized HOMO and LUMO of (a-b) Cu-MOFs and (c-d) Zn-MOFs.

**Supplementary Figure 12.** HOMO–LUMO orbitals gap of Cu-MOFs, Zn-MOFs and Cu_2_Zn_1_-MOFs.
